# Perceived Concerns of Azeri Menopausal Women in Iran

**DOI:** 10.5812/ircmj.11771

**Published:** 2014-05-05

**Authors:** Sevil Hakimi, Masoumeh Simbar, Fahimeh Ramezani Tehrani

**Affiliations:** 1 Department of Midwifery and Reproductive Health, Faculty of Nursing and Midwifery, Shahid Beheshti University of Medical Sciences, Tehran, IR Iran; 2 Safe Motherhood Research Center, Department of Midwifery and Reproductive Health, Shahid Beheshti University of Medical Sciences, Tehran, IR Iran; 3 Reproductive Endocrinology Research Center, Research Institute for Endocrine Sciences, Shahid Beheshti University of Medical Sciences, Tehran, IR Iran

**Keywords:** Menopause, Particulate Matter, Iran

## Abstract

**Background:**

Menopause is a complex biological phenomenon which is affected by socio-cultural and physiological factors. These factors may cause different experiences and concerns for menopausal women from different countries and even among women of diverse ethnic groups living in the same country. The signs and symptoms of menopause are exacerbated by the negative attitude of menopausal women and their deep concerns.

**Objectives:**

We aimed to explore self-experienced concerns of Iranian menopausal women residing in Tabriz.

**Materials and Methods:**

Phenomenological approach was used for analyzing the participants’ experiences and perceptions about menopause. Data were collected through 18 semi-structured in-depth interviews; carried out from February to July 2012. Participants were menopausal women aged between 46-57 years who experienced menopause no more than 4 years before the interview. All interviews were audio recorded and transcribed verbatim. Constant comparative analysis of the data was conducted using MAXQDA 10.

**Results:**

Participants’ concerns were classified into 4 main themes, including: inability, aging, isolation, and healthiness.

**Conclusions:**

Understanding these concerns might contribute to the enrichment of the existing literature by providing evidences from a different culture, assisting to design effective supportive strategies, planning training programs and appropriate infrastructures for women to improve their quality of life during the menopausal period.

## 1. Background

Menopause is a physiological event which characterized by apparently falling levels of estrogen and can lead to the development of symptoms such as hot ﬂashes, night sweats, vaginal dryness, mood swings, libido decline, insomnia, lethargy, fatigue, irritability, anxiety, depression, heart palpitations, and joint pain (1). The symptoms of menopause are typically multifactorial in nature (2), and are different regarding women’s culture, society, education and economic condition (3-8). In fact, the experience of menopause is unique to each individual, and its meaning differs among women (9).

Menopausal symptoms have shown differences among women in different countries, among women of different origins living in the same country, and among women born in the same place living in different countries (8). For example, the frequency of hot flashes ranges from 80% among American women, to 12% in Japanese, with none at all among the Mayans (10-12). In the Western part of the world the menopause is often considered as the negative part of life, and described as a deﬁciency syndrome (3). Furthermore, the medical literature is mostly dominated by biomedical opinions about symptoms and loss of well-being.

Some women experience a profound sense of loss at menopause (e.g. loss of maternal role, youth or beauty) which may lead them to feel that life has lost its purpose (13). A qualitative research in Turkey showed that getting old, loss of sexual interest and vasomotor symptoms were negative experiences in menopausal women. Emotional instability or irritability is among the prevalent complaints and lack of family support seemed to worsen mood swing (14).

Understanding these concerns might contribute to the enrichment of the existing literature by providing evidences from a different culture, and helping to design effective supportive strategies and appropriate infrastructures for women to improve their lives during the menopausal period.

Overall menopausal symptoms are found to be less common in societies where menopause is viewed as a positive rather than a negative event (15, 16). Women with negative attitudes towards the menopause experience more frequent and severe symptoms during the menopausal transition (17, 18).

A quantitative research to address specific aspects of the menopausal transition showed that Iranian women consider menopause as a natural event. But exposure to a new situation and deal with the phenomenon depends on their attitudes. Iranian women’s concerns are about complications such as osteoporosis, sexual problems and aging (19). They also experience positive and negative changes during the menopause, and their negative experiences are associated with the severity of symptoms (20).

In spite of extensive research on menopausal symptoms and its psychosocial aspects, little is known about the personal meaning or view of the menopausal transition as experienced by Iranian women. Furthermore, there is not any exclusive study about concerns of menopausal women regarding their culture. The current phenomenological study explored the lived experienced concerns of menopausal Azeri women.

## 2. Objectives

This study was a part of larger phenomenological study that explored physical and psychosocial experiences of the menopausal women. The main objective of this research was to explore the menopausal women’s concerns.

## 3. Materials and Methods

A hermeneutic phenomenological approach, as described by Van Manen (1990), was used to explore the women’s concerns about menopause. Hermeneutic phenomenology aims to “uncover the structure of lived experience”. It is important to understand the lives of individuals in their own context by taking into account individuals’ life experiences and meanings derived from those experiences. Furthermore, to gain a full understanding of the meaning or essence of an experience, the experience needs to be described and interpreted (21, 22).

The ethics committee of Shahid Beheshti University of Medical Sciences approved the study beforehand. Each participant was informed about the purpose of the research, its procedures, risk, benefits, and the voluntary nature of the participation. Furthermore, the subjects have the right to stop the research at any time. The informed consent was given to the participants and explained in detail for illiterate women.

### 3.1. Participants

The context of this study was set in Tabriz (a city in the northwest of Iran with population around 1.5 million). A sample of 18 menopausal women was recruited by health care providers in the urban health centers over six months (from February to July 2012). They were a heterogeneous group from different educational levels, occupational and marital statuses. Samples were selected through the purposeful sampling method. In this type of sampling, researchers recruit sample respondents with a rich experience of the phenomenon (menopause) and the ability and tendency to express their experiences (19). No participant refused the interview.

 We defined menopause according to the World Health Organization classification as a condition of absence of spontaneous menstrual bleeding for more than 12 months, for which no other pathologic or physiologic cause could be determined. We selected those who met our eligibility criteria, which included lacking a history of endocrine disorders, hysterectomy, oophorectomy, and psychological disorders. Those with menopausal age less than 40 years or reached menopause more than 4 years before recruitment were rejected from the study.

### 3.2. Data Collection

Face-to-face, in-depth, and semi-structured interviews were performed for data collection, and all interviews were audiotaped. Participants were given the opportunity to talk about the study-related issue. All of interviews were conducted at the urban health centers. For achieving the maximum variance of participants, we selected 10 health centers that were located in different places with respect to socio-economic conditions.

 The interviews were conducted by a single researcher (first author), in Azeri language and took place in a quiet private room, and no one was present except the researcher and participant during the interviews. The interview guide had three questions, which were provided to collect data about experienced concerns of menopausal women. The interviews were continued until no new information or opinions were obtained about the interview topics.

 The average length of interviews was about 30-50 minutes. All audiotaped interviews were transcribed verbatim. A pilot study on three women had been conducted to refine the interview guide beforehand. The interviewer asked three main questions to help the interviewees get focused. The questions were as follows: “What did you feel when you became aware that you were going through menopause?”, “What was your main complain during your menopause?”, and “Were you concerned about these complains?” All the written transcripts were compared with the audiotapes for accuracy, and any errors in transcription were corrected. After becoming familiarized with the raw data, we classified and coded them.

### 3.3. Data Analysis

With regard to Van Manen’s research activities about the selective or highlighting approach, the statements or phrases that seem particularly insightful into the phenomenon was used in this study. Data were analyzed concurrently with the data collection, using thematic analysis as described by van Manen (22). To gain further perspectives and themes, researchers read the transcripts several times and highlighted essential statements and phrases related to menopausal women`s concerns as thematic statements.

In order to bring the meanings to the surface, the researchers frequently wrote, rewrote, went into depth, and reviewed the findings several times. On several occasions during the research/analysis process, the researchers ‘stepped back’ and looked at the whole context again, and analyzed how each part has contributed to the whole (21). Finally, the emerging themes were classified. MAXQDA- 10 was used to assist data analysis and organization.

### 3.4. Trustworthiness

The trustworthiness of the findings was enhanced using Lincoln and Guba’s criteria: credibility, transferability, dependability and confirmability (23). To achieve credibility, we used maximum variance among participants, i.e. the participants had different levels of education, socio-economic class, and so on. Furthermore, analyzed transcripts were returned to three participants in order to validate the data and ensure trustworthiness of the described experiences.

One of the mentioned participants was illiterate, and the recorded interview was explained exactly and read for her word by word. Also, bracketing was used to increase credibility (21). Dependability was guaranteed by continues and complete documentation of researcher activities related to data gathering and analysis. For enhancing confirmability, the researchers of the team reviewed the interviews and coded them. The concordance between coding was 80%. To ensure transferability, the authors described exactly the essential research context and its assumptions in order that the other researcher can conduct the similar projects.

## 4. Results

Eighteen semi-structured and in-depth interviews were conducted. The mean age of participants was 52.11 ± 2.9 years (age range: 46-57 years). The characteristics of participants are shown in (Table 1).

**Table 1.** Participant’s Demographics

Variables	Number	Percentage
**Age, y**		
40-50	4	22.2
More than 50	14	77.7
**Education**		
Illiterate	6	33.3
Primary and guidance school	8	44.4
High school & diploma	3	16.6
Higher education	1	5.5
**Current marital status**		
Married	16	88.8
Widow	2	11.1
**Employment status**		
Housewife	14	77.7
Employee	4	22.2
**Number of children**		
2-4	12	66.7
5-6	6	30.3
**Menopausal period**		
Less than 2 year	9	50
2-4 year	9	50

Thematic analysis showed 4 main concerns, composed of “inability”, “elderly”, “isolation”, and “losing wellness”. Findings are shown in (Table 2).

**Table 2.** A Summary of Findings

Inability
Keeping an independent life Performing religious ritual
Performing daily housework
Memory loss
**Aging**
Future of aging
Elder feature
**Isolation**
Irritable mood
Urine malodor
**Loosing wellness and healthiness**
Osteoporosis
High Blood pressure, Diabetes and Hyperlipidemia
Musculoskeletal problems
Mental problems
Cardio vascular disease, Myocardial infarction
Alzheimer

### 4.1. Concerns About Inability

All of the participants were worried about their “inability” in the future. Menopausal women were concerned about “inability” to live an independent life, performing the religious rituals, performing daily housework, and forgetfulness.

#### 4.1.1. Inability to Live an Independent Life

All of the participants were worried about losing their autonomy and being a burden to their children and other family members. Muscle and joint pain was cited as the leading sign of the sense of “inability”.

“I’m afraid of osteoporosis; I love to travel. I hate being dependent on others and I am afraid to be a burden to my family; I like to manage my family, if one day I lose my autonomy, I will break… ” (53 years old with academic education, married).

“I have severe pain in my knees and back; I am worried about becoming paralyzed. If I am unable to move and become immobile, who will do my housework? Nowadays, nobody helps the others…. I’m afraid of becoming a burden to under obligation of my children… “(47 years old, illiterate, married).

#### 4.1.2. Inability in Performing the Religious Rituals

About half of participants disclosed that they could not perform their daily prayers and other religious rituals due to urinary incontinence. The urine is considered impure (Najes) in the Islamic rules, and therefore, it invalidates the daily prayer. Muslims are commanded to pray with clean clothes. “I have this urine problem; I go to the toilet normally but when I laugh, cough and sneeze a few drops of urine come out. I change my underwear continuously. I can’t pray because I’m unclean and impure … I am very concerned about it…” (49 years, primary school education, married).

#### 4.1.3. Inability in Performing Daily Housework

Less than half of the participants suffered from premature fatigue. They mentioned that they could not perform their daily housework as usual because they get tired very fast. “I can’t do my chores, because I get tired too fast. Whenever I can’t finish my daily chores I get nervous, but I can’t do them right and conduct them correctly, I like my house to be clean and organized. Sometimes I am preoccupied about the future. What will happen if I lose the capacity to clean my house, what would become of my life? (49 years, primary school education, married).

#### 4.1.4. Memory Loss

About half of the women were worried about their episodic memory loss and indicated that their memory was impaired after menopause. They mentioned that they had problems in recalling people’s name, messages, telephone numbers and addresses, and they cannot plan for their daily life. “My memory condition worries me. I can’t remember some of the words, I have no memory of anything to be able to talk with people, and I can't hold a conversation anymore” (52 years, illiterate, married). "I am telling my children: don’t give me anything because I may misplace it; When someone asks me something, I forget it” (51 years, Diploma, widow).

### 4.2. Concern About Aging

Some women were concerned about getting older after the onset of menopause. In their view, menopause has a close relation with aging and it is the start of senility. Two sub-themes that covered this theme were “future” and “appearance”.

#### 4.2.1. Future

Some women expressed their concern about the future and aging. “I’m worried about my future and health. What I’m doing in the future? I feel my body shrinking down, and my health worsens day after day. What will happen to my health? ” (46 years, diploma, married).

#### 4.2.2. Appearance

Half of the participants were concerned about their face and skin. “After menopause the wrinkles are appearing in my face. Every time I glance in the mirror, I see wrinkles, and I remember my youth and the years I lost” (51 years, diploma, married). But majority of participants mentioned that the aging is not associated with menopause. "Menopause is not getting old. Getting old depends on our feelings and spirit. If a woman exercises and travels; she can stay young even 20 years after menopause or even more. I hope to get old after the age of 80 or 90" (54 years, diploma, widow, and employee). “The wrinkles are a part of my face; they are not related to menopause. This is a normal process which is the result of aging not menopause” (57 years, elementary school education, married, housewife).

### 4.3. Concerns About Isolation

The sub-themes of this experience were isolation for mood swing and malodor urine.

#### 4.3.1. Irritable Mood

Some participants expressed concerns for mood swings, irritability, depression and their tendency to bring about loneliness that affected them after menopause.

“I am always sad and depressed. My children and my husband are very kind, and I haven’t any problem in my life. But I feel really sad since I became menopause. Sometimes I feel my children get tired of me. They don’t understand me. They say, you are always impatient … I know that if I got irritated, nobody would like me… people must be kind and patient. I know this and I am afraid that nobody can tolerate my mood, and I will end up alone” (49 years, primary school education, married, housewife).

#### 4.3.2. Malodor Urine 

Some participants disclosed that they have urinary incontinence and are concerned of urine malodor and isolation. ”I suffer from incontinency. I think that I smell like urine. I’m worried. If I smell continuously, nobody likes me. Nobody wants to speak with me. Nobody wants to come to my house (52 years, illiterate, married).

### 4.4. Losing Well-being and Healthiness

One of the participants’ concerns was expressed around health problems, including osteoporosis, blood sugar and lipids, muscle skeletal problems, psychological problems, myocardial infarction and forgetfulness.

#### 4.4.1. Osteoporosis

A number of women were worried about osteoporosis. ‘’I heard about osteoporosis. My mother suffered from osteoporosis; my sisters were affected also. I’m afraid that one day I am affected by osteoporosis just like my mother and sisters (53 years, Academic education, married, and employee).

#### 4.4.2. Rising blood Sugar and Lipids

 Some participants mentioned that menopause was a predisposing factor for diabetes and elevation of blood lipids. “I have diabetes. My physician told me that it’s due to the menopause. I am concerned that my blood pressure and lipids might be raised. I am afraid of high blood pressure (54 years, elementary school education, married, and housewife).

#### 4.4.3. Musculoskeletal Problems

Half of the participants stated that they had suffered from joint pain and muscle stiffness, especially in the mornings. “When I wake up, I have lots of muscle and joint pains. My body felt extremely stiff and achy and one or two hours must be passed till I feel better by exercise, and I should do some exercises. I think why I am always sick? What is my disease? (49 years, illiterate, married, housewife).

#### 4.4.4. Psychological Problems

Some of the participants were concerned about their psychological problems. “I cannot sleep well since I became menopause. My doctor warned me about harmful effects of sleeplessness on my heart and blood pressure. Do I have psychological problems? Do I have a serious disease? (49 years, illiterate, married, housewife).

#### 4.4.5. Alzheimer

Some of the participants expressed their concerns about Alzheimer and forgetfulness after menopause. “I forget everything; I forget the order (Rakat) of my prayer. I’m worried about getting Alzheimer. I think my brain is hanging (48 years, elementary school education, married, and housewife).

"Sometimes, my children’s friends call and leave a message. After getting off the phone, I forget who called. I forget the massages. Last week, I couldn’t find my house after getting off the taxi. I was very afraid. I told myself: What should I do? A Few moments later, I remembered my home address. I am afraid that one day I lose my mind and get Alzheimer".

## 5. Discussion

This study is the first qualitative research that conducted to explore Azeri menopausal women`s concerns and hidden fears associated with menopause. This study demonstrated that the main concerns of women were “inability,” “aging,” “isolation,” and “healthiness and well-being”.

These women were worried about losing their independency, and having a productive life, outward appearance and the inner nature of aging, bereaving their communication with others because of their irritable and changing mood, as well as bad smell secondary to their urinary incontinence, and finally suffering from osteoporosis, cardiovascular diseases and cancers. Their fear of inability was also aggravated by experiencing pain in their joints and muscles. They were worried about pain worsening that may lead to their inability to perform daily activities and leave them to be a burden for others.

The concern about functional ability may be related to feelings of losing autonomy and decreasing self-esteem in addition to the viewing menopause as an ageing process. It is demonstrated that cancerous people concern about being a burden for other family members, and experience a feeling of losing their identity by increasing dependency to others (24).

There are very limited facilities and social services for elders in Iran (24). So, they were afraid of inability and future of elderly. Regarding the out-of-pocket payment in our health system (25) and respecting the decrease in the fertility rate (26) and fading of close ties between family members in recent decades, women are afraid of inability and a future that nobody can take care of them.

The participants mentioned the inability to perform religious rituals resulted from urinary incontinence as a concern during menopause. It is well documented that religion has beneficial effects on adjustment to physical, social, and existential aging (27). Religion endows the elders a sense of meaning, mastery, security, belongingness, identity, and continuity (28). The findings of the present study are consistent with another study in Iran that showed ostomy patients were worried about their religious rituals, which had been disrupted by colostomy complications (29).

It is essential to be clean and free of any fecal and urine material for praying in Islam. The religion has a strong relation with Iranian culture and daily praying is one of the most important and vital parts of Iranian women's life. So, women's concern about inability for praying was expected.

The concern about inability for performing housework resulted from premature fatigue. Management of house chores is among the main responsibilities of Iranian women and failure to conduct them induces the sense of inability. It is demonstrated that Jordanian menopausal women concern about fatigue (9), perhaps because of the similar responsibilities. The participants were concerned about forgetfulness. Complains about memory loss and cognitive abilities occurs during the transitional period from premenopausal period to menopause (30, 31). Forgetfulness has a negative effect on the quality of life (32). Memory loss often threatens perceptions of security, autonomy and being a meaningful member of the society (33).

Our findings showed some of the participants were worried about elderly. Their concern was related to deteriorating health and "feature and their face." While others considered aging as a natural process which should not be worried about it. In another research in Iran, women concerned about aging (19). Similar findings have been documented in other studies from different countries, i.e. some menopausal women were concerned about elderliness, and others believed that getting older is not related to the menopause rather is a normal part of life, and the sign of maturity (14, 34).

 Our findings indicated that the minority of women who worried about their appearance came from middle social class according to their education and socio-economic class. Delanoe et al. in a qualitative study noted that “concern about seeming” expresses mainly from the middle social class women (8). It seems that women with low education and socio-economic situation are not concerned about their appearance. Women expressed their concern about social isolations due to irritability, mood swings, depression, and the like.

The influence of endocrine function on mood swing of menopausal women needs to be researched. Probably, psychological factors, lifestyle, body image, interpersonal relationships, role, and socio-cultural factors affect mood swing in menopausal women (35). The participants were worried that their family might reject them due to their irritability. Women felt that their close family members did not understand them, and therefore, they felt uneasy to talk about what they were experiencing during menopause. Depression and lack of tolerance to noisy environments were also reported by the participants. They were afraid to stay isolated from their children and grandchildren.

In a qualitative study, some women thought they should seek help if they experience a swinging temper because it seemed so intolerable (36). In another study, the participants’ irritable state seemed to be interfering with their family relationships, and some of the participants expressed their need for support and understanding from those around them (14). Thus, it appears that family support and understanding, will help in reducing women's stress during this period.

Women expressed their concern about isolations due to odorous urine resulted from incontinency. They concerned about their relationships with their family members and friends, which were endangered by urinary incontinence and might lead to the loss of social relations, social support and identity. Fear of the smell of the urine is reported very often by women who experienced urinary incontinence (37). In addition, women who experienced urinary incontinence reported that they felt that their social identity was in danger (38).

Our participants also worried about their physical and mental health. Iranian women`s concern about osteoporosis, and blood pressure was reported in another study (19). Thai middle-aged women expressed vulnerability to poor health related to menopause, osteoporosis, and managing stress (39). Some of the qualitative researches showed menopausal women looked upon their symptoms related to the menopause both as consequences of hormonal changes and as a part of natural ageing (39, 40). The women participated in the present study, attributed all of physical and mental changes to menopause. One of the probable cause of their beliefs was the negative attitude toward menopause due to lack of information. With increasing middle-aged population and life expectancy in Iran, developing and performing special training and programs in a primary health care system is necessary.

This study was a qualitative study, and the findings can provide a deep understanding of women’s perceptions and experiences about menopause; findings which could not be achieved through quantitative studies. Variety in sampling was an advantage of the current study. Participants of the study belonged to different socio-economic backgrounds. However, this study has some limitations. Menopause is a private and sensitive issue in life of the Azeri women, and speaking about such topics is not easy. Although the authors tried to develop an intimate atmosphere during the interviews, the participants may not completely be comfortable to reveal their feeling and experiences. Voluntary participation made a frame for exclusion of the experiences of those who did not wish to participate in the study for any reason. Also, all participants were selected from an urban community of Tabriz. Therefore our findings do not reflect menopausal experiences of rural communities or other Iranian ethnicities.

Menopausal women experience four main concerns of inability, aging, isolation and losing wellness and healthiness. It seems that our participants felt insufficient support by their family members. Regarding the recent trend of Iranian population toward elderliness, integrating the comprehensive educational and care program for menopausal women and their family members in primary health care system and developing appropriate infrastructure for elderly people seems to be necessary.
